# Distinct Translational Control in CD4^+^ T Cell Subsets

**DOI:** 10.1371/journal.pgen.1003494

**Published:** 2013-05-02

**Authors:** Eva Bjur, Ola Larsson, Ekaterina Yurchenko, Lei Zheng, Valentina Gandin, Ivan Topisirovic, Shui Li, Carston R. Wagner, Nahum Sonenberg, Ciriaco A. Piccirillo

**Affiliations:** 1 Department of Microbiology and Immunology, McGill University, Montreal, Canada; 2 FOCIS Centre of Excellence, Research Institute of the McGill University Health Centre, Montreal, Canada; 3 Department of Biochemistry, and Goodman Cancer Research Centre, McGill University, Montreal, Canada; 4 Department of Chemistry, University of Minnesota, Minneapolis, Minnesota, United States of America; The Jackson Laboratory, United States of America

## Abstract

Regulatory T cells expressing the transcription factor Foxp3 play indispensable roles for the induction and maintenance of immunological self-tolerance and immune homeostasis. Genome-wide mRNA expression studies have defined canonical signatures of T cell subsets. Changes in steady-state mRNA levels, however, often do not reflect those of corresponding proteins due to post-transcriptional mechanisms including mRNA translation. Here, we unveil a unique translational signature, contrasting CD4^+^Foxp3^+^ regulatory T (T_Foxp3+_) and CD4^+^Foxp3^−^ non-regulatory T (T_Foxp3−_) cells, which imprints subset-specific protein expression. We further show that translation of eukaryotic translation initiation factor 4E (eIF4E) is induced during T cell activation and, in turn, regulates translation of cell cycle related mRNAs and proliferation in both T_Foxp3−_ and T_Foxp3+_ cells. Unexpectedly, eIF4E also affects Foxp3 expression and thereby lineage identity. Thus, mRNA–specific translational control directs both common and distinct cellular processes in CD4^+^ T cell subsets.

## Author Summary

Regulatory T cells expressing the nuclear protein Foxp3 are essential for the control of immune responses towards self and foreign antigens. Genome-wide gene expression studies have defined canonical signatures of T cell subsets. However, changes in mRNA levels often do not reflect those of corresponding proteins due to post-transcriptional mechanisms including mRNA translation. In Bjur *et al*., we discovered a unique translational signature, which distinguishes immunosuppressive Foxp3^+^ regulatory T from inflammatory Foxp3^−^ T cells and establishes proteomes and functions in T cell subsets. We also show that cell activation or growth factors increase the translation of eukaryotic translation initiation factor 4E (eIF4E), which induces proliferation in both T cell subsets. Unexpectedly, eIF4E also affects Foxp3 expression and can drive lineage identity. Thus, distinct translational control directs both common and distinct cellular processes in CD4^+^ T cell subsets.

## Introduction

Regulation of gene expression is a multi-step process involving transcriptional, post-transcriptional and post-translational mechanisms. Recent studies have revealed that only 30–40% of steady state protein levels correspond to steady-state mRNA levels and identified mRNA translation as the principal post-transcriptional mechanism [1], [2]. Furthermore, several studies have documented that changes in steady-state mRNA expression-profiles frequently do not correspond to changes in the proteome [3]–[6]. Thus, studies of the translatome (i.e. those mRNAs that are being translated) can potentially help to explain biological processes beyond standard profiling of mRNA levels.

CD4^+^ T helper (Th)-cell lineage differentiation is defined by expression of specific transcription factors required for subset identity [7]. Foxp3 is a master-switch transcription factor impacting lineage commitment by driving the intra-thymic differentiation of natural CD4^+^Foxp3^+^ regulatory T (T_Foxp3+_) cells, a critical mediator of immune self-tolerance and prevention of excessive inflammatory responses [8], [9]. In the absence of Foxp3, CD4^+^ T cells can differentiate into a spectrum of inflammatory effector subsets. Furthermore, Foxp3 expression can be up-regulated in CD4^+^ T cells to generate induced T_Foxp3+_ (iT_Foxp3+_) cells [10] *in vitro* and *in vivo*. Genome-wide expression profiles using steady-state mRNA samples have defined canonical “T_Foxp3+_ gene expression signatures” that distinguish primary resting or activated T_Foxp3+_ from CD4^+^Foxp3^−^ non-regulatory T (T_Foxp3−_) cells [11]–[15]. In contrast, studies of mRNA translation in T cells are limited but suggest that T cells augment mRNA-translation and induce translation of specific mRNAs upon activation [16]–[18]. However, such studies compared non-activated to activated total CD4^+^ T cells, used non genome-wide approaches and/or immortalized cell lines and are thus limited in scope. Hence, the contribution of mRNA translation to establishment of the proteome in different T cell subsets is still largely unknown. We therefore asked whether translational control contributes to establishment of the proteomes in T_Foxp3+_ and/or T_Foxp3−_ cells.

Here, we report the first genome-wide study on translational control in primary CD4^+^ T_Foxp3+_ and T_Foxp3−_ cell subsets directly *ex vivo* and post-activation *in vitro*. We reveal substantial mRNA specific quantitative and qualitative differences in the translatome between primary CD4^+^ T cell subsets. Remarkably, these translationally regulated genes were not previously identified in genome-wide studies of steady-state mRNA and therefore provide hereto unknown information on gene expression programs in T cell subsets. We further identified distinct translational control of the eIF4E-mRNA as a mechanism regulating proliferation in both T_Foxp3+_ and T_Foxp3−_ cells. Surprisingly, modulation of eIF4E activity also affects T cell lineage identity. Thus, CD4^+^ T cell subsets exhibit common and specific translational programs that orchestrate expression of genes that direct fundamental cellular processes.

## Results

### Genome-wide analysis of CD4^+^ T cell subset translation

**Figure 1:**
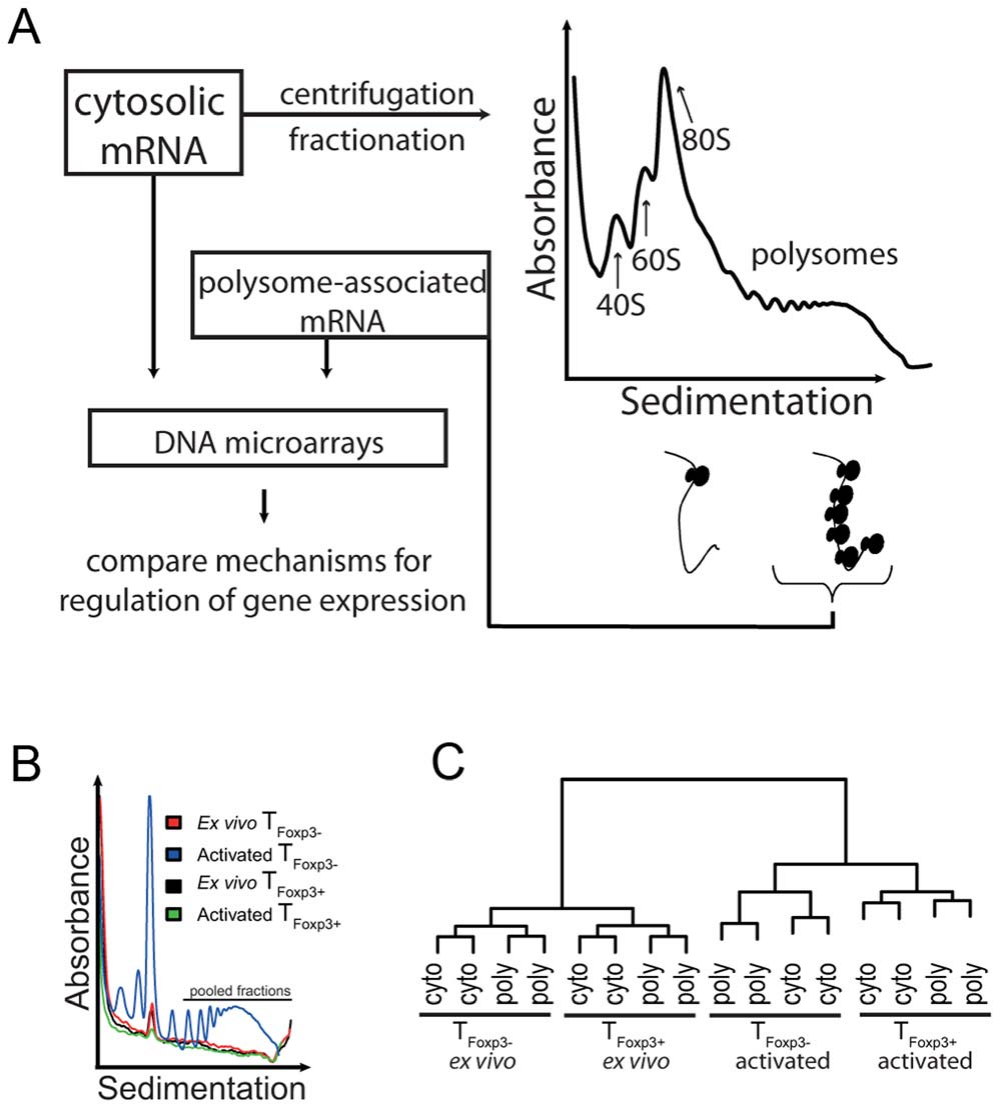
Genome-wide analysis of translationally regulated mRNAs in primary CD4^+^ T cell subsets. (a) Cytosolic mRNA was extracted and probed directly with DNA microarrays or processed using the polysome preparation technique where mRNAs are sedimented on a sucrose gradient and separated based on the number of ribosomes they associate with. Fractions containing mRNAs that engage ≥3 ribosomes were pooled and probed with microarrays to quantify mRNA levels. (b) Polysome UV-tracings from *ex vivo* and *in vitro* activated T_Foxp3+_ and T_Foxp3−_ cells. Shown is the UV absorbance (254 nm) as a function of sedimentation. The large peak corresponds to the 80S ribosome peak and was used to align the polysome profiles so that fractions containing ≥3 ribosomes could be pooled from each sample. The part of the polysome profile that was pooled and used as the polysome-associated mRNA sample is indicated. (c) Assessment of data set quality. Shown is a dendrogram from a hierarchical clustering of all included samples (using Pearson correlations). Samples that are more similar cluster together. Cyto – cytosolic mRNA; poly – polysome-associated mRNA.

Translation is mainly regulated at the initiation step, during which ribosomes are recruited to the mRNA [19]. Efficiently translated mRNAs are therefore associated with a larger number of ribosomes than poorly translated mRNAs. Consequently, an approach to enrich for mRNAs being translated is based on poly(ribo)some preparations where mRNAs from cytoplasmic extracts are sedimented according to the number of ribosomes they bind (Figure 1a).

To determine whether CD4^+^ T cell subsets regulate gene expression at the level of mRNA translation, we prepared cytosolic and polysome-associated (with ≥3 ribosomes) mRNA from either primary T_Foxp3+_ or T_Foxp3−_ CD4^+^ cells (i.e. CD4^+^ and GFP^+^ or GFP^−^ cells from Foxp3-GFP reporter knock-in mice) directly *ex vivo* or 36 h post *in vitro* activation. Isolation of polysome-associated mRNA in sufficient quantities was technically challenging because T_Foxp3+_ cells represent a scarce population (5–10% of total CD4^+^ T cells), and T_Foxp3+_ and T_Foxp3−_ cells are relatively inactive transcriptionally and translationally *ex vivo*. Consequently, the UV-absorption profiles of polysomes from T_Foxp3+_ cells and *ex vivo* T_Foxp3−_ cells were below the detection limit except for the 80S ribosome peak (Figure 1b). The 80S peak was therefore used to align all polysome RNA preparations to assure that fractions with mRNAs carrying the same number of ribosomes (≥3) were pooled for each sample. Affymetrix GeneChips were then used to quantify genome-wide cytoplasmic and polysome-associated mRNA levels. We assessed the reproducibility of the procedure by comparing gene expression data across all genes and samples using Pearson correlations (Figure 1c). The replicates clustered according to activation state followed by cell type and RNA origin indicating that, despite low mRNA amounts, high quality, reproducible data were obtained.

### Translatomes of Foxp3^−^ and Foxp3^+^ CD4^+^ T cells are distinct

**Figure 2:**
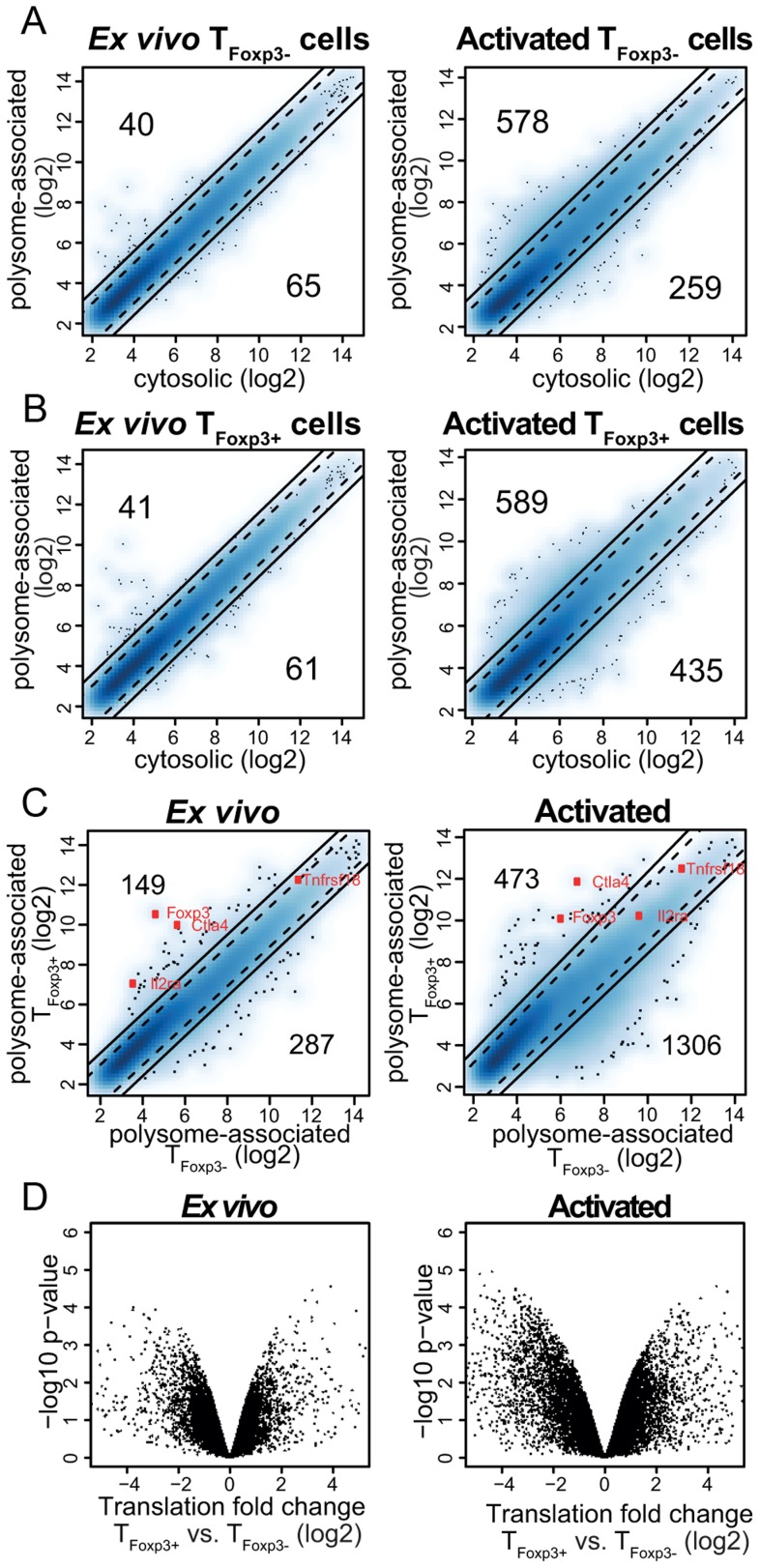
A translational signature that discriminates T_Foxp3+_ and T_Foxp3−_ cells. (a–b) Polysome-associated mRNA levels differ from cytosolic mRNA levels in primary CD4^+^ T cell subsets *ex vivo* and post-activation. Shown are density scatter plots of polysome-associated vs. cytosolic mRNA data (a blue scale from light to dark represents increasing local density of data points; outliers are indicated as dots) for T_Foxp3−_ cells (a) and T_Foxp3+_ cells (b) at the *ex vivo* and the activated condition. The solid and dotted lines indicate a >3-fold and >2-fold difference, respectively, in the density scatter plot. The number of mRNAs that show a >3-fold difference in each direction is indicated. (c) Substantial differences in levels of polysome-associated mRNA between T_Foxp3+_ and T_Foxp3−_ cells. Density scatter plots (as in a–b) compare polysome-associated mRNA data between T_Foxp3+_ and T_Foxp3−_ cells in both the *ex vivo* and *in vitro* activated conditions. A few genes known to be differentially expressed between T_Foxp3+_ and T_Foxp3−_ cells are indicated (*Foxp3, Ctla4, Il2ra* [CD25] and *Tnfrsf18* [GITR]). As expected the differential expression of *Il2ra* is lost upon activation. (d) Differential translation in T_Foxp3+_ vs. T_Foxp3−_ cells as identified with anota-RVM *ex vivo* and post *in vitro* activation. Significances (i.e. the −log10 p-value from the anota analysis used to identify differential translation) are compared to log2 translational fold changes (after correction for cytosolic mRNA levels).

To assess whether studies of polysome-associated mRNAs provide new information regarding gene expression in CD4^+^ T cells, we compared polysome-associated to cytosolic mRNA levels in T_Foxp3−_ cells directly *ex vivo* or post activation *in vitro*. While polysome-associated mRNA levels largely resembled those of cytosolic mRNAs in the *ex vivo* condition (although many mRNAs showed moderate differences [2–3-fold]), abundant and dramatic differences (>3-fold) were observed in activated T_Foxp3−_ cells (Figure 2a). Similarly, in T_Foxp3+_ cells (Figure 2b) differences between levels of polysome-associated and cytosolic mRNAs occurred primarily in the activated condition. Thus, polysome-associated and cytosolic mRNA profiles differ indicating that steady-state mRNA signatures may not faithfully reflect corresponding protein levels for many genes.

As T_Foxp3+_ and T_Foxp3−_ cells were isolated and treated identically, we expected comparable levels of polysome-associated mRNAs after correcting for differences in cytosolic mRNA levels (i.e. that translation would be regulated uniformly across T cell subsets). To examine this we first compared data from polysome-associated mRNA between T_Foxp3+_ and T_Foxp3−_ cells (Figure 2c). This analysis showed that T_Foxp3+_ and T_Foxp3−_ cells vary substantially in terms of which mRNAs are more abundant in polysomes, particularly in activated cells. However, to identify those mRNAs that show differential translational activity, data from polysome-associated mRNAs must be corrected for cytosolic mRNA data to exclude a contribution from e.g. transcription or RNA-stability. We performed such correction using anota [20], [21] and, unexpectedly, found large differences in translational activity of specific mRNAs, especially between activated T cell subsets (Figure 2d). After adjusting the p-values for multiple testing, we found that while differences in translation were modest *ex vivo* (∼20 mRNAs with a Benjamini-Hochberg false discovery rate [FDR] <30%, corresponding to a nominal p-value<0.004), there were substantial differences in translation between activated T cell subsets (∼200 and 500 mRNAs were translationally activated or suppressed, respectively, in activated T_Foxp3+_ as compared to T_Foxp3−_ cells [FDR<15%]). These data strongly suggest that translational control plays an important role in regulating gene expression programs in T_Foxp3+_ and T_Foxp3−_ cell lineages.

### A unique translational signature discriminates activated CD4^+^ T cell subsets

Although we identified specific mRNAs that showed both qualitative and quantitative differences in translational activity between activated T cell subsets, this signature may overlap with previously described steady-state mRNA signatures and hence not shed light into unknown aspects of T cell gene expression. This possibility arises because while we used cytosolic mRNA levels to correct levels of polysome-associated mRNAs, previous studies measured whole cell steady-state mRNA levels (which also reflect nuclear mRNA levels). We therefore compared the activated T cell translational signature to data from 5 independent studies of steady-state mRNA levels [11]–[15], and focused our analysis on comparisons between T_Foxp3+_ and T_Foxp3−_ cells isolated *ex vivo* or activated *in vitro* (Figures S1, S2). Although iT_Foxp3+_ cells only partially recapitulate the T_Foxp3+_ steady-state mRNA signature, we also determined whether this signature overlapped with the translation signature (Figure S2) [11], [15], [22]. To assess the overlap with steady-state mRNA signatures we calculated the percentage of mRNAs that were translationally regulated and exhibited differential mRNA levels in any of the studies of steady-state mRNA levels. Only 11% of the mRNAs were shared (Figure S1) and only 5% were identified in at least two steady-state mRNA signatures (7 additional comparisons confirmed this pattern, Figure S2). Although we expect that there will be differences between cytosolic (present study) and whole-cell (previous studies) steady-state mRNA signatures we wanted to validate that the observed distinct translational signature was not entirely driven by a very small overlap between these. We therefore performed the same analysis but compared our signature from cytosolic mRNA to previous datasets on steady-state mRNA. In contrast to the translational signature the signature from cytosolic mRNA showed a considerable overlap (47% or 32% were shared between the present cytosolic and at least one or two steady-state mRNA signatures, respectively) – indicating that the lack of overlap between the translational signature and previous steady-state signatures is not due to that we studied cytoplasmic mRNA. Similar comparisons to the translational signature from *ex vivo* cells were hampered by that few genes were differentially translated (Figure S3). Thus, the newly identified translational signature discriminating activated CD4^+^ T cell subsets is unique.

### A modular organization of translation in activated CD4^+^ T cell subsets

**Figure 3:**
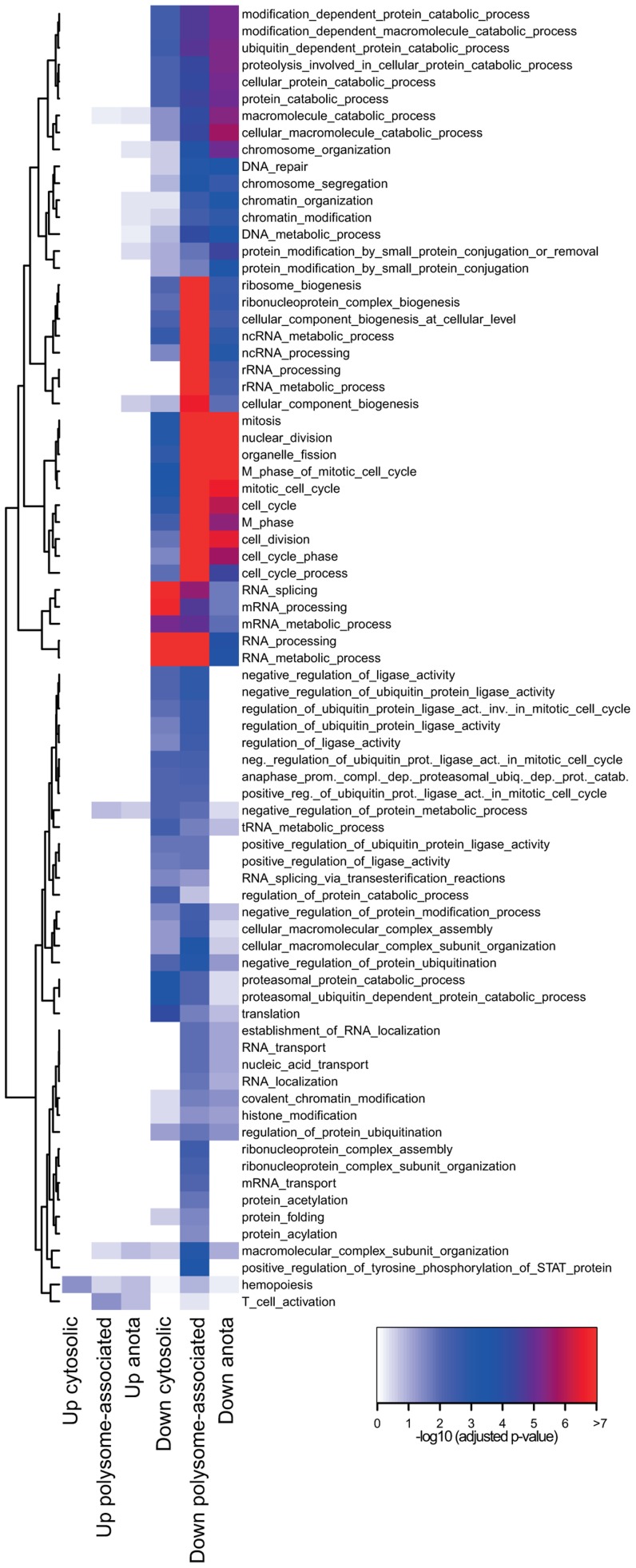
Distinct modular translational control between activated CD4^+^ T cell subsets. Graphical representation of the enrichment analysis within subsets of mRNAs identified as differentially expressed (up in T_Foxp3+_ cells or down in T_Foxp3+_ cells) in data from cytosolic mRNA, polysome-associated mRNA and as differentially translated by anota (after correction for cytosolic mRNA levels). The subsets are shown as columns and the rows represent cellular functions that were enriched. The colour scale represents −log10 p-values (adjusted for multiple testing) for the enrichment. All p-values that were <10e-7 were set to 10e-7.

**Figure 4:**
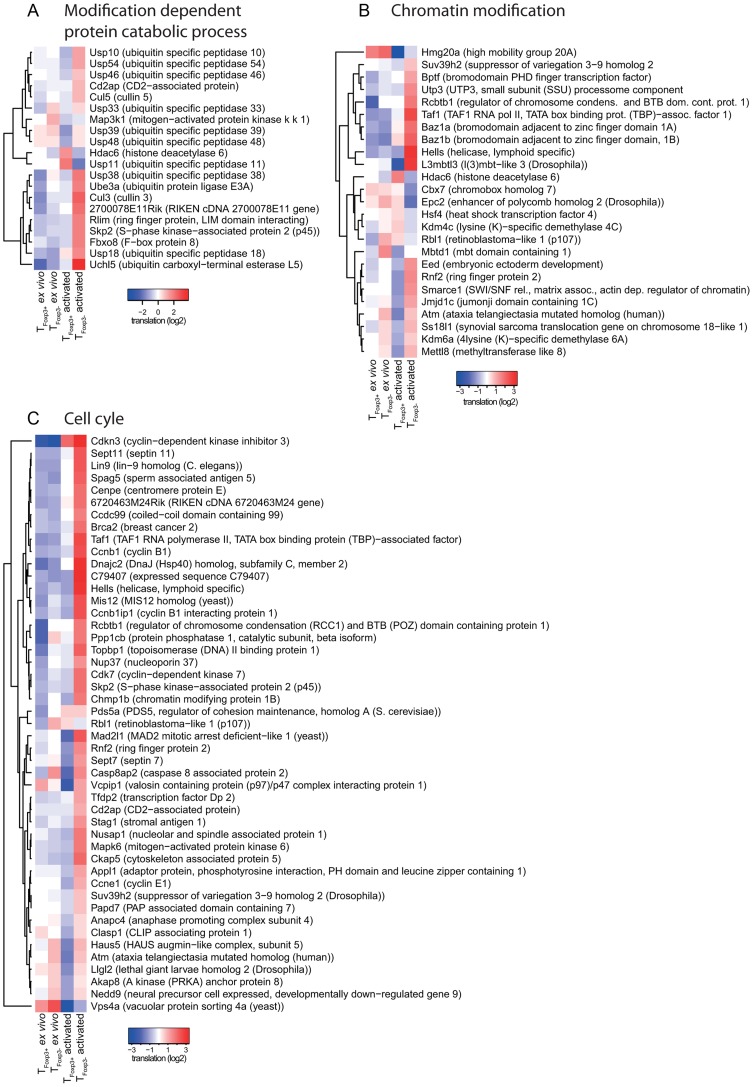
Translationally regulated mRNAs encode proteins are involved in ubiquitination, chromatin modification, or cell cycle pathways. Translational activity (from anota after correction for cytosolic mRNA levels) in T_Foxp3+_ and T_Foxp3−_ cells *ex vivo* and post *in vitro* activation for individual mRNAs belonging to ubiquitination (a), chromatin modification (b) or cell cycle (c) pathways is shown. The colour scale represents translational activity in log2 scale.

Gene expression programs are commonly viewed as being “modular” where each module consists of several co-regulated genes that control specific cellular functions and several studies indicate the existence of such modules at the post-transcriptional level [23]–[25]. We therefore determined the functional relationship between mRNAs that are translationally regulated in a T cell subset-specific manner. To assess whether there was an overlap of cellular functions targeted by differential translation or cytosolic mRNA levels, we also identified mRNAs that were differentially expressed using data obtained from cytosolic mRNA. As a control, we studied mRNAs that were differentially expressed using data obtained from polysome-associated mRNA (functions regulated at the translational level [significant after anota analysis] should also be regulated using data from polysome-associated mRNA). We separated the resulting mRNAs into those that were activated or suppressed in T_Foxp3+_ cells as compared to T_Foxp3−_ cells and sought for significantly enriched biological functions in each subset (Figure 3). Few functions were enriched among mRNAs that were translationally more active in activated T_Foxp3+_ cells, whereas translationally suppressed mRNAs were highly functionally related. When comparing to the enrichment analysis for cytosolic mRNA data, several functions were primarily regulated at the level of translation including ubiquitination, chromatin modification and cell cycle. Such functions were also identified as regulated by translation (FDR<0.05) using an alternative gene set enrichment approach (GAGE) [26]. To further examine these functions, we collected all differentially translated mRNAs annotated to the identified cellular functions and compared their translational activity across all studied conditions (Figure 4a–4c). For each function there was a strong signature regarding both the number of mRNAs involved and the magnitude of differential translation between activated T_Foxp3+_ and T_Foxp3−_ cells. For the cell cycle cluster, the profile was uniform as most mRNAs were translationally suppressed in activated T_Foxp3+_ cells as compared to activated T_Foxp3−_ cells. The chromatin modification and ubiquitination clusters contained both translationally activated and suppressed mRNAs in activated T_Foxp3+_ cells as compared to activated T_Foxp3−_ cells, indicating complex regulation of these functions via translational control. Thus, the translational signature contrasting activated T cell subsets is enriched for mRNAs whose encoded proteins participate in distinct cellular processes.

### Differential translation of the eIF4E-mRNA in activated T_Foxp3+_ and T_Foxp3−_ cells contributes to establishment of their proteomes

**Figure 5:**
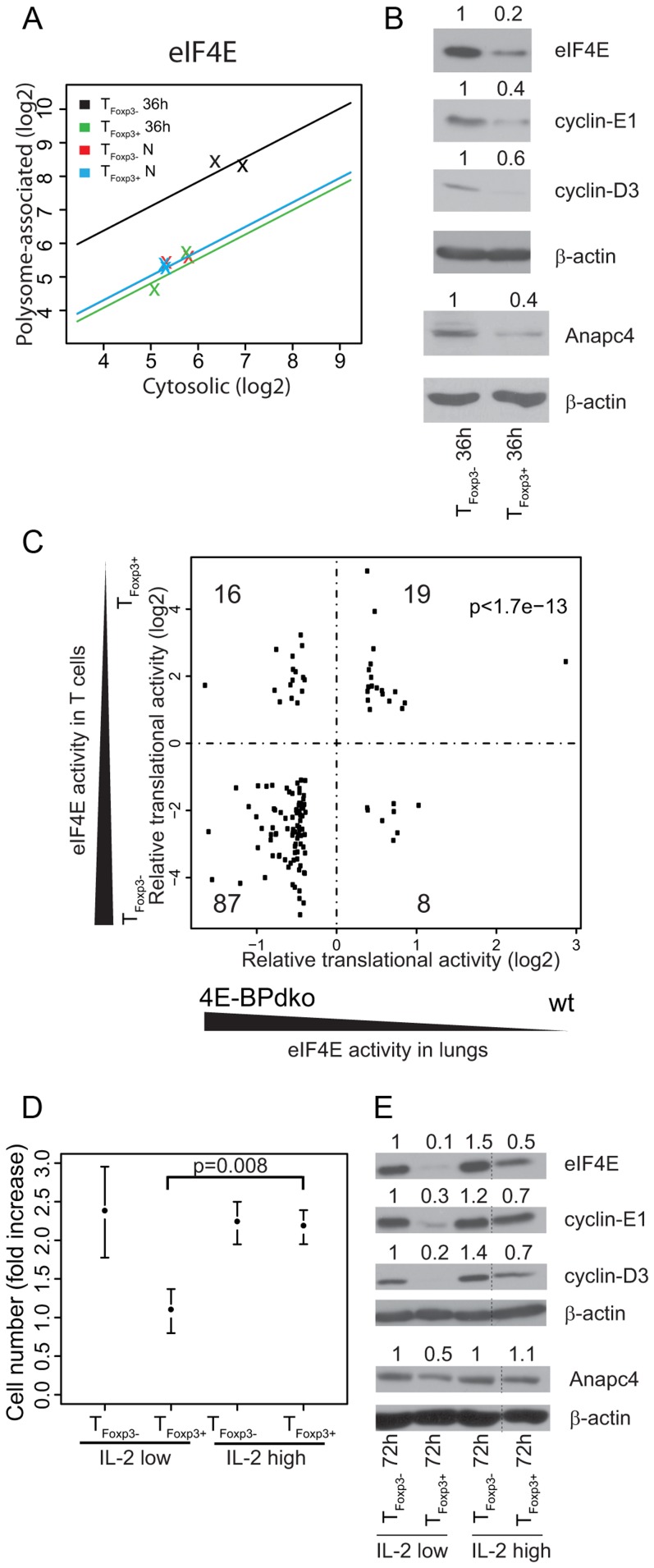
Differential levels of eIF4E between T_Foxp3+_ and T_Foxp3−_ cells partly explain their translational signature and correlate with CD4^+^ T cell subset proliferation. (a) eIF4E is translationally more active in activated T_Foxp3−_ cells as compared to T_Foxp3+_ cells. Shown is the cytosolic mRNA level (x-axis) vs. the polysome-associated mRNA level (y-axis) for each condition; T_Foxp3+_ N (blue) and T_Foxp3−_ N (red) – *ex vivo* cells; T_Foxp3+_ 36 h (green) and T_Foxp3−_ 36 h (black) – *in vitro* activated cells. The lines indicate the regressions used by anota to correct the polysome-associated mRNA level for the cytosolic mRNA level. (b) Activated T_Foxp3−_ cells express higher protein levels of eIF4E, cyclin-E1, cyclin-D3, and Anapc4 as compared to activated T_Foxp3+_ cells. Shown are western blots from T_Foxp3+_ and T_Foxp3−_ cells activated for 36 hours. Densitometry was used to quantify protein levels and obtained levels were normalized to β-actin (the normalized values were related to T_Foxp3−_ 36 h which was set to 1 and are indicated above each lane). (c) Identification of an eIF4E responsive module in the activated T cell translational signature. Fold changes from differentially translated mRNAs from the activated T cell translational signature that also showed a fold change difference for translation in lungs from 4E-BPdko mice are plotted. The number of mRNAs in each quadrant is shown. (d) High IL-2 concentration induces proliferation in T_Foxp3+_ cells. Cell numbers were counted when plated and after 72 h of culture with low (100 U/ml) or high (1000 U/ml) IL-2 concentrations. The fold increase in cell number was calculated and associated means and standard deviations (n = 3) are shown. Welch's two sample t-test was used to compare T_Foxp3+_ cells cultured under different IL-2 concentrations. (e) High IL-2 concentration induces eIF4E expression in T_Foxp3+_ cells. Shown are western blots of total protein extracts probed with antibodies for eIF4E, cyclin-E1, cyclin-D3, and Anapc4 in T_Foxp3+_ and T_Foxp3−_ cells activated as described in (d). Densitometry was used to quantify protein levels and obtained levels were normalized to β-actin (the normalized values were related to T_Foxp3−_ 72 h IL-2 100 U/ml which was set to 1 and are indicated above each lane; lanes between lanes 3 and 4 in (e) were spliced out but all shown lanes are from the same gel).

Surprisingly, we identified eIF4E as translationally suppressed in activated T_Foxp3+_ cells as compared to activated T_Foxp3−_ cells. eIF4E is the rate-limiting translation initiation factor that binds to the mRNA 5′-cap structure to recruit mRNA to the ribosome [19]. Activated T_Foxp3+_ cells showed a 5-fold translational suppression of eIF4E as compared to activated T_Foxp3−_ cells (Figure 5a). Consistently, the levels of eIF4E protein were higher in activated T_Foxp3−_ than in activated T_Foxp3+_ cells (Figure 5b). eIF4E dramatically regulates translation of mRNAs which encode proteins participating in various cellular processes including cell cycle [27]–[29], apoptosis [30] and innate immunity [31] but only modestly affects global protein synthesis. Thus, parts of the activated T cell translational signature could be mediated by an activation-induced disparity in eIF4E levels between T_Foxp3+_ and T_Foxp3−_ cells.

eIF4E activity is repressed by the eIF4E-binding proteins (4E-BPs) which compete with eIF4G for binding to eIF4E. Kim *et al.* recently measured genome-wide translational activity in lungs from wild type (WT) and 4E-BP1/2 double-knockout mice (4E-BPdko) [32]. 4E-BPdko mice would hence be expected to show increased eIF4E activity as compared to WT. Although the impact of increased eIF4E activity likely differs between T cells and lungs, the eIF4E translational signature could nonetheless be partly conserved, reflecting the central role of eIF4E in cellular function. We therefore compared the translational signature contrasting activated CD4^+^ T cell subsets to the signature contrasting 4E-BPdko and WT lung to determine if part of the activated translational signature could be accounted for by differences in eIF4E activity. Strikingly, more mRNAs showed similar regulation between the two studies than distinct regulation (Figure 5c, binomial test p-value = 1.7e-13). Notably, differential eIF4E levels, as observed between T_Foxp3+_ and T_Foxp3−_ cells, had larger impact on translation as compared to presence or absence of 4E-BPs (compare log2 fold changes in Figure 5c between T cells and lungs). Thus, a part of the translational signature from activated T cell subsets can be explained by differences in eIF4E levels.

Whereas 87 of the similarly regulated mRNAs were translationally regulated in a manner that paralleled the activity of eIF4E (eIF4E-sensitive), 19 mRNAs showed translational suppression following eIF4E activation. These 19 mRNAs could either represent the noise in the comparison or reflect a phenomenon observed in several studies of translational control downstream of eIF4E that is likely caused by secondary effects [27], [28]. To assess the phenotypic consequence of increased eIF4E levels, we identified enriched functions among the 87 encoded proteins whose translation paralleled eIF4E activity, and identified 17 biological functions, including cell cycle and ubiquitination (Table S1). Further analysis revealed that 25% of the mRNAs in the eIF4E signature were related to the cell cycle and that ubiquitination partly overlapped with the cell cycle cluster as close to one fourth (22%) of the cell cycle genes were annotated also to the ubiquitination system. Indeed, proteins translated from eIF4E-sensitive cell cycle related mRNAs were more highly expressed in activated T_Foxp3−_ cells as compared to activated T_Foxp3+_ cells (Figure 5b; cyclin E1 is part of the translational signature; translation of Anapc4 paralleled eIF4E activity also in mouse lungs; and cyclin-D3 is eIF4E sensitive [29]). Thus, the translational signature differentiating activated CD4^+^ T cell subsets exhibits functional and mechanistic modularity.

### IL-2 mediated induction of eIF4E and proliferation in T_Foxp3+_ cells

Although T_Foxp3+_ cells are suppressive following *in vitro* activation they are, unlike T_Foxp3−_ cells, anergic to T cell receptor (TCR)-induced proliferation. However, despite their anergy *in vitro*, T_Foxp3+_ cells can expand under homeostatic or inflammatory settings *in vivo* [33]. Because our translational signature from *in vitro* activated T cells compared suppressive and anergic T_Foxp3+_ cells to non-suppressive and proliferating T_Foxp3−_ cells the signature will reflect both suppressive activity and anergy. Consistently, although we identified an enrichment of cell cycle related genes as translationally suppressed in T_Foxp3+_ cells activated *in vitro* these only represented ∼11% of the mRNAs that were translationally suppressed and only a minute fraction of the ∼200 genes that were translationally activated (Figure 4c) – indicating that most of the translational signature is related to other biological processes differentiating activated T_Foxp3−_ and T_Foxp3+_ cells. Nevertheless, the correlation between eIF4E level, translational activation of proliferation-related genes (Figure 5c, Table S1) and proliferation raises the possibility that eIF4E may control proliferation in both T_Foxp3+_ and T_Foxp3−_ cells. To assess the relationship between eIF4E level and T_Foxp3+_ cell proliferation, we used a condition where the *in vitro* anergy of T_Foxp3+_ cells to TCR signals is rescued by relatively higher doses of exogenous IL-2 [34]. A higher concentration of IL-2 induced proliferation of T_Foxp3+_ cells to a level similar to that observed for T_Foxp3−_ cells (Figure 5d) and strikingly also induced higher eIF4E protein levels (Figure 5e). The increase in eIF4E level was accompanied by increased synthesis of cell cycle related proteins from eIF4E sensitive mRNAs (Figure 5e). Thus, IL-2 abrogates the anergy in T_Foxp3+_ cells, which is associated with increased eIF4E levels and translation of eIF4E sensitive mRNAs. In aggregate, these data indicate that eIF4E may control expansion of both T_Foxp3+_ and T_Foxp3−_ subsets.

### eIF4E induced proliferation in T cell subsets is independent of 4E-BPs

**Figure 6:**
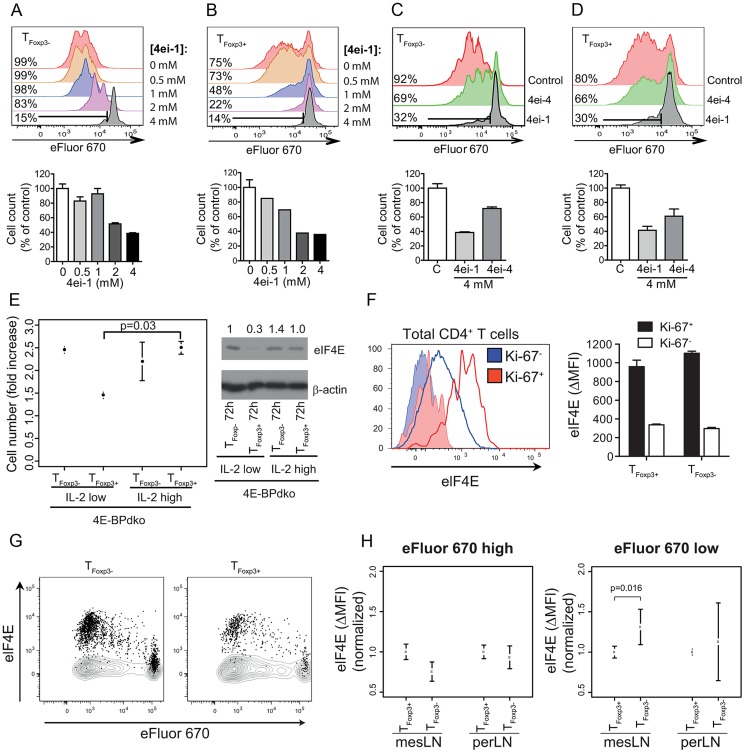
eIF4E controls proliferation in T cell subsets. (a) Inhibition of eIF4E activity suppresses T_Foxp3−_ cell proliferation. eFluor 670-labeled T_Foxp3−_ cells were IL-2/TCR-activated for 72 h in the presence of increasing concentrations of the eIF4E inhibitor 4ei-1 (K_d_ = 0.80 µM). Proliferation was determined under each condition by eFluor 670 dilution assessed by flow cytometry (upper panel). The effect on proliferation was also assessed by comparing cell counts after 72 h under each condition (lower panel; the control was set to 100%). (b) Inhibition of eIF4E activity abrogates IL-2-mediated reversal of anergy in T_Foxp3+_ cells. IL-2/TCR-activated eFluor 670 labelled T_Foxp3+_ cells were cultured in the presence of 4ei-1, and proliferation was determined as described in (a). (c–d) IL-2/TCR-activated eFluor 670 labelled T_Foxp3−_ cells (c) or T_Foxp3+_ cells (d) were cultured in the presence of 4ei-1 or 4ei-4. Proliferation was determined under each condition as described in (a). (a–d) Representative histograms from 4 independent experiments are shown (upper panels; the percentages of proliferating cells are indicated). Means and standard deviations of cell counts from 4 independent experiments are shown (lower panel). (e) Induction of T_Foxp3+_ cell proliferation occurs independently of signalling through 4E-BPs. 4E-BPdko T_Foxp3+_ and T_Foxp3−_ cells were plated and counted as described in (Figure 5d), and the fold increase in cell number was calculated and associated means and standard deviations (n = 2) are shown. Welch's two sample t-test was used to compare 4E-BPdko T_Foxp3+_ cells cultured under different IL-2 concentrations. Also shown is a western blot of total protein extracts probed with antibodies for eIF4E in 4E-BPdko T_Foxp3+_ and T_Foxp3−_ cells. Densitometry was used to quantify protein levels and obtained levels were normalized to β-actin (the normalized values were related to T_Foxp3−_ 72 h IL-2 100 U/ml which was set to 1 and are indicated above each lane). (f) Ki-67 and eIF4E co-expression in total CD4^+^ T cells isolated directly *ex vivo* from lymph nodes (left panel). Quantification of eIF4E expression is shown as Δ (eIF4E *vs.* isotype control) mean fluorescent intensity (MFI). Filled histograms represent staining with an isotype control. Quantification of eIF4E expression (ΔMFI) in Ki-67^+/−^ T_Foxp3−_ and T_Foxp3+_ cells isolated directly *ex vivo* (right panel, mean and standard deviation is indicated, n = 3). (g–h) eFluor 670-labeled T_Foxp3−_ or T_Foxp3+_ cells adoptively transferred into separate TCR β−/− mice were isolated from mesenteric (mes) and peripheral (per) lymph nodes (LN) followed by measurement of eFluor 670 and eIF4E expression four days post transfer. (g) Representative dot plots (n = 3) of T_Foxp3−_ and T_Foxp3+_ cell proliferation relative to eIF4E expression in mesLN. Staining with an isotype control are shown as contour plots. (h) Quantification of eIF4E expression (ΔMFI) in cells that have (eFluor 670 low) or have not (eFluor 670 high) undergone cell division (means and standard deviations are indicated after per experiment normalization to T_Foxp3+_ cells, n = 4–6). P-value (Welch two sample t-test) is indicated.

It was therefore important to examine whether increased eIF4E activity is necessary for induction of T_Foxp3+_ and/or T_Foxp3−_ cell proliferation *in vitro*. To this end, we used an eIF4E inhibitor – the pro-nucleotide 4ei-1 (Figure S4), which inhibits binding of eIF4E to the mRNA cap structure and thereby selectively reduces eIF4E activity and eIF4E sensitive translation. 4ei-1 is a stable, non-toxic, pro-nucleotide that, when activated intracellularly by HINT, binds to eIF4E with a K_d_ of 0.80 µM [35]. Strikingly, 4ei-1 suppressed proliferation and accumulation of activated T_Foxp3−_ and T_Foxp3+_ cells in a dose-dependent manner (Figure 6a–6b) without affecting viability or IL-2R expression (Figure S5a–S5b). To assess the selectivity of the drug response, we treated cells with a structurally-related eIF4E inhibitor, 4ei-4 (Figure S4), that has a 10-fold lower affinity for eIF4E (K_d_ = 7.5 µM) as compared to 4ei-1. The inhibitory effect of 4ei-4 on T_Foxp3+_ and T_Foxp3−_ cell proliferation was substantially lower as compared to 4ei-1 (Figure 6c–6d). The apparent increased anti-proliferative effects of eIF4E inhibition in T_Foxp3+_ cells compared to T_Foxp3−_ cells (Figure 6a–6b) could be related to the differential eIF4E protein levels in these T cell-subsets (Figure 5e) and/or differential uptake of 4ei-1. Thus induced eIF4E activity is necessary for proliferation in both T_Foxp3+_ and T_Foxp3−_ cells *in vitro*.

Although our data indicated that modulation eIF4E activity occurs through translational activation of the eIF4E mRNA leading to induced eIF4E protein levels (Figure 5a–5b), regulation of eIF4E also occurs via 4E-BPs. The 4E-BPs are inhibitors of eIF4E downstream of mTORC1 and are inactivated by mTOR signalling [19]. To examine whether signalling through the 4E-BPs was also necessary for induction of proliferation, we TCR-activated T_Foxp3+_ or T_Foxp3−_ cells from 4E-BPdko/Foxp3-GFP mice in the presence of low or high IL-2 concentrations. High IL-2 concentration augmented proliferation and correlated with increased expression of the eIF4E protein also in T_Foxp3+_ cells from 4E-BPdko mice (Figure 6e). Moreover, the proliferative potential of T_Foxp3+_ and T_Foxp3−_ cells of either genotype was comparable under similar conditions (compare Figure 5d and Figure 6e). The lack of contribution from the 4E-BPs could be explained by their sustained inactive state following *in vitro* activation-induced signalling through the mTOR pathway. Consequently, 4E-BP deficiency will not further affect eIF4E activity. Thus, translational activation of the eIF4E-mRNA, independent of signalling via the 4E-BPs, is necessary for proliferation of T_Foxp3+_ or T_Foxp3−_ cells.

Next, we sought to validate that eIF4E levels are also associated with proliferation of T_Foxp3+_ and T_Foxp3−_ cells *in vivo*. For this we first established a flow cytometric approach to quantify eIF4E levels during T cell subset proliferative responses and evaluated it *in vitro*. Such analysis confirmed that *in vitro* proliferation of both T_Foxp3+_ and T_Foxp3−_ cells is associated with higher expression of eIF4E and indicated that T_Foxp3+_ and T_Foxp3−_ cells proliferate and induce eIF4E expression with different kinetics (Figure S6). Flow cytometric analysis of cells isolated *ex vivo* confirmed that proliferating (Ki-67^+^) CD4^+^ T cells exhibit higher eIF4E expression as compared to non-proliferating (Ki-67^−^) cells (Figure 6f left panel), irrespective of T cell subset (Figure 6f right panel). Finally we examined the relationship between eIF4E expression and proliferation *in vivo*. To this end, T_Foxp3+_ or T_Foxp3−_ cells were adoptively transferred into TCRβ^−/−^ recipient mice and isolated from mesenteric (mes) or peripheral (per) lymph nodes (LN) 4 days post-transfer. Both proliferating (eFluor670^low^) T_Foxp3+_ and T_Foxp3−_ cells expressed higher eIF4E levels than non-proliferating (eFluor670^high^) cells (Figure 6g). Intriguingly, and consistent with our findings *in vitro* (Figure S6), the eIF4E level was higher in proliferating (eFluor670^low^) T_Foxp3−_ cells as compared to T_Foxp3+_ cells in mesLN but not in perLN (Figure 6h). This suggests that microenvironmental factors such as the inflammation in mesenteric sites may selectively enhance eIF4E-induced T cell subset expansion. Thus eIF4E level correlates with T_Foxp3+_ and T_Foxp3−_ cell proliferation *in vivo*.

### eIF4E mediated control of T cell subset identity

**Figure 7:**
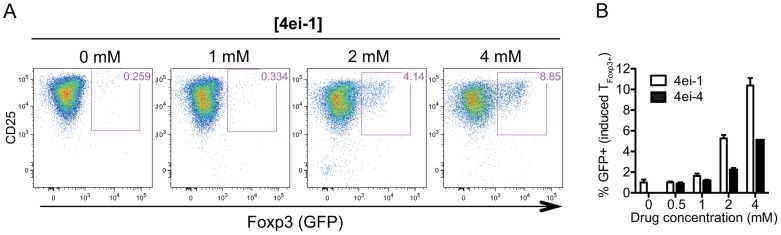
Inhibition of eIF4E activity results in spontaneous induction of Foxp3 expression in activated T_Foxp3−_ cells. T_Foxp3−_ cells were IL-2/TCR-activated for 72 h in the presence of increasing concentrations of 4ei-1 or the control pro-drug 4ei-4 in undifferentiating conditions, and Foxp3 expression (i.e. GFP) was assessed by flow cytometry. (a) Representative density plots from experiments using T_Foxp3−_ cells cultured in the presence of 4ei-1 from 4 independent experiments are shown. (b) Percentage Foxp3^+^ cells following treatment with 4ei-1 or 4ei-4 (shown are means and standard deviations, n = 4).

Whereas mTOR deficiency blocks differentiation into Th1, Th2 or Th17 cells under respective polarizing conditions, *in vitro* activation of mTOR deficient T_Foxp3−_ cells induces Foxp3 expression and a suppressive phenotype [36]. Furthermore, inhibition of mTOR in T_Foxp3−_ cells induces Foxp3 expression accompanied by T_Foxp3+_-like steady-state mRNA and microRNA expression profiles [37]. Given that we identified eIF4E as a component responding to *in vitro* activation, we asked whether eIF4E activity affects subset identity. To this end, TCR-activated T_Foxp3−_ cells from Foxp3-GFP mice were treated with 4ei-1 and Foxp3 expression was measured by GFP fluorescence. Strikingly, there was a dose-dependent induction of Foxp3 expression in activated T_Foxp3−_ cells upon inhibition of eIF4E activity using 4ei-1 under undifferentiating conditions (Figure 7a). A similar experiment using 4ei-4 resulted in substantially less Foxp3 induction suggesting that strong inhibition of eIF4E activity is required for T_Foxp3+_ cell differentiation (Figure 7b). Collectively, these data pinpoint to the modulation of eIF4E activity as a key component that affects T cell subset identity.

## Discussion

A functional immune system relies on controlled and coordinated induction, and rapid termination of immune responses to avoid erroneous or excessive triggering of pro-inflammatory responses. In this regard, translational control of gene expression appears advantageous as compared to transcriptional control as it provides a fast mode of action that does not require *de novo* mRNA synthesis. Accordingly, a number of individual mRNAs encoding proteins involved in both innate and adaptive immunity are regulated at the translation step. Expression of IRF7 is normally translationally suppressed to avoid faulty activation of the interferon response [31]; T cell production of the chemokine RANTES/CCL5 is dependent on the transcription factor RFLAT-1 whose expression is translationally regulated [38]; translational suppression of cytokine production is a key mechanism by which self-reactive T cells are kept anergic [39]. Here we show that regulation of mRNA translation plays a central role in the orchestration of genetic programs in T_Foxp3+_ and T_Foxp3−_ lineages. Activation of T_Foxp3+_ and T_Foxp3−_ cells leads to selective qualitative and quantitative changes in translational activity of specific mRNAs. Notably, genes in the newly identified translational signature have not been uncovered in previous comparisons of T_Foxp3+_ and T_Foxp3−_ cells using steady-state mRNA and therefore represent hereto unknown aspects of CD4^+^ T cell biology. Qualitative and quantitative changes in translation are more pronounced upon T cell activation, in agreement with the notion that translation is often regulated during cellular responses that require rapid and coordinated control of protein expression. However, this does not exclude that individual mRNAs are translationally regulated in the resting state, although the number of these mRNAs appears to be modest as compared to that observed upon activation.

Coordinated regulation of groups of functionally related mRNAs has been postulated to be a common mechanism by which cellular functions are regulated [23]. Here we identified eIF4E as a target for post-transcriptional regulation which, in a modular fashion, activates translation of a set of cell cycle related genes - thereby further exemplifying the complexity of how post-transcriptional circuits affect cellular functions [23]. While the T cell activation-associated dramatic increase in translation was previously suggested to be linked to eIF4E activity [40], we show that eIF4E induction is necessary for proliferation of both T_Foxp3+_ and T_Foxp3−_ cells *in vitro* and that eIF4E level correlates with cell proliferation *in vivo*. We thereby provide important insights into regulation of proliferation of T cell subsets. The translational signature also involved individual genes which may be part of yet undefined post-transcriptional modules but with previously established functions in T cell biology. Foxo1 and Foxo3 [41], [42], both have essential roles in the stability of Foxp3-dependent T_Foxp3+_ cell lineage commitment [43]. Interestingly, we found translational activation of the Foxo3 mRNA (4-fold) in T_Foxp3+_ as compared to T_Foxp3−_ cells in the activated condition (Figure S7) indicating that translational control of specific mRNAs may be important for expression of proteins regulating T cell lineage commitment.

A developmental relationship exists between various Th cell effector lineages, suggesting a high degree of functional plasticity which enables cells to switch from one lineage to another [44]. Cytokines including IL-2, TGF-β1 and IL-10 influence the induction or stability of Foxp3 expression in iT_Foxp3+_ or natural (n) T_Foxp3+_ cells thereby influencing T_Foxp3+_ cell fate and the type of immune response. Surprisingly but in agreement with previous studies on mTOR signalling, we identified eIF4E, a downstream target of mTOR signalling, as affecting T cell lineage identity. However, our data do not exclude that other downstream targets of mTOR also contribute to cell lineage identity or that the effects are indirect (e.g. as a result of inhibition of proliferation during cell activation). Thus, further studies will be needed to address whether the effects of eIF4E on cell lineage identity are direct or indirect.

Collectively our data favour a model whereby eIF4E levels could be dynamically regulated in response to changes in the local inflammatory environment thereby providing a direct link between the extracellular micro-environment, gene expression and biological responses.

## Materials and Methods

### Mice

GFP-Foxp3 knock-in (ki) mice have been described previously [13] and were kindly provided by A. Y. Rudensky (now at Memorial Sloan-Kettering Cancer Centre, NY). For the experiments with 4E-BP deficient T cells, GFP-Foxp3ki mice were crossed to 4E-BPdko mice. TCRβ−/− mice are αβT cell deficient due to the absence of the gene encoding the TCR βchain. Mice were housed and bred under specific pathogen free conditions according to Canadian Council on Animal Care (CCAC)-approved institutional guidelines at the animal facility of the Department of Microbiology and Immunology; McGill University. Female mice 6–12 weeks old were used for the study.

### Isolation of CD4^+^ T cell subsets and preparation of cytosolic and polysome-associated RNA

Cells isolated from lymph nodes and spleens were stained with PE conjugated CD4 antibody (GK1.5, eBioscience, San Diego, CA) and MACS purified. Thereafter T_Foxp3+_ and T_Foxp3−_ cells were sorted based on CD4 and GFP-Foxp3 expression using a FACSAria to obtain cell populations of high purity (>97%). For the naïve cells all buffers and media were supplemented with cycloheximide (Sigma, St. Louis, MO) (100 µg/ml). Cycloheximide immobilizes ribosomes on the mRNA and enables separation of polysome-associated RNA. For the activated samples cells were activated for 36 h with plate bound CD3 and CD28 antibodies (BD Bioscience) (5 µg/ml) in the presence of recombinant hIL-2 ([100 U/ml]: a kind gift from the Surgery Brach, NCI/NIH). Cycloheximide (100 µg/ml) was added to the medium at the end of the culture. Cytosolic and polysome-associated RNA were prepared directly *ex vivo* or post-activation *in vitro* as described previously [27] and labelled for probing with microarrays using the Ovation Pico WTA system (NuGEN) according to the manufacturer's instructions. All experiments were performed in biological duplicates. For cells isolated directly *ex vivo*, RNA from two experiments was pooled for each sample.

### Data analysis

Data were extracted and normalized using rma implemented in the R package “affy” (www.r-project.org) using updated probe set definitions (ENTREZ_GENE) [45]. Integrity of samples was assessed using 5′ to 3′ ratios and the comparability of the arrays by scaling factors. The reproducibility was assessed by correlation analysis using both Spearman and Pearson correlations in R and visualized using the hclust function in R. All these analyses confirmed good data quality. We used anota-RVM [20] to identify differential translation and applied the following stringent filtering for gene selection to assure correct linear models in anota: slopeP = 0.05; maxSlope = 1.5; minSlope = (−0.5); deltaP = 1; deltaPT = 1 (as defined in the anotaPlotSigGenes function in anota [21]). We used Benjamini-Hochberg multiple testing adjusted p-values (false discovery rates [FDR]) as a cut off for differential translation (FDR<30% for *ex vivo* cells and FDR<15% for activated cells). RVM was also applied to identify differentially expressed mRNAs between T_Foxp3+_ and T_Foxp3−_ cells using data from polysome-associated mRNAs and cytosolic mRNA data from the activated condition. The resulting p-values were corrected using the Benjamini-Hochberg multiple testing adjustment method and an FDR <15% was used as cut off. We used GO::Termfinder [46] to identify enriched cellular functions within subsets of differentially regulated mRNAs that were upregulated in T_Foxp3+_ cells (123 unique mRNAs from the cytosolic mRNA analysis; 226 unique mRNAs from the polysome-associated mRNA analysis; and 251 unique mRNAs that were translationally regulated from anota) or upregulated in T_Foxp3−_ cells (404 unique mRNAs from the cytosolic mRNA analysis; 666 unique mRNAs from the polysome-associated mRNA analysis; and 504 unique mRNAs that were translationally regulated from anota) and collected those functions that showed: >2-fold enrichment; at least 10 annotated and regulated mRNAs; and a FDR <1%. For identification of the eIF4E signature we down-loaded the data set with the accession number GSE17406 from the Gene Expression Omnibus (GEO) and used anota to identify differential translation. mRNAs that were differentially translated in the T_Foxp3+_ vs. T_Foxp3−_ comparison and showed >1.3-fold difference in the GSE17406 data set were collected and compared to identify an overlapping eIF4E translational signature (the analysis was robust at more restrictive fold changes). The binomial p-value for rejecting the NULL hypothesis (no eIF4E signature) was calculated in R. This dataset has been deposited at the Gene Expression Omnibus (GEO) accession GSE45401.

### T cell proliferation

T_Foxp3+_ and T_Foxp3−_ cells were activated for 72 h with plate-bound anti-CD3 and -CD28 antibodies in the presence of 100 U/ml or 1000 U/ml of recombinant human IL-2 (rhIL-2). Cells were counted before plating and at the end of the culture to determine the fold-increase in cell number. Cell viability was assessed either using a trypan blue or eFluor780 Fixable Viability Dye (eBioscience, San Diego, CA) exclusion assays. For the inhibition of eIF4E activity *in vitro*, T_Foxp3+_ and T_Foxp3−_ cells were stained with the Cell Proliferation Dye eFluor 670 (eBioscience, San Diego, CA) and activated as described above in the presence of rhIL-2 (1000 U/ml) and in the presence or absence of selective inhibitors of mRNA cap structure-binding to eIF4E: 4ei-1, a prodrug (pronucleotide phosphoramidate) of 7Bn-GMP (K_d_ of 0.80 µM), or its control 4ei-4, a prodrug of 7Me-GMP, which has a 10- fold lower affinity for eIF4E than 7-Bn-GMP (K_d_ = 7.5 µM). When assaying T cell proliferation *in vivo* total CD4^+^ T cells, isolated from LNs of GFP-Foxp3 ki mice, were stained directly *ex vivo* with a V450-conjugated Ki-67 antibody (B56, BD Biosciences, Missisauga, ON) and a primary eIF4E antibody or an isotype control (Abcam, Cambridge, MA) followed by staining with a PE-conjugated secondary antibody (Abcam, Cambridge, MA). ΔMFI for eIF4E was calculated by subtracting the MFI value for the isotype control from that obtained with the eIF4E antibody. For the experiments involving TCRβ−/− mice, congenic (Ly5.1+) T_Foxp3−_ (CD4^+^CD25^−^) and T_Foxp3+_ (CD4^+^CD25^+^) cells were MACS purified from GFP-Foxp3 ki mice based on CD4 and CD25 expression, subsequently stained with the eFluor 670 Cell Proliferation Dye and adoptively transferred into separate TCRβ−/− recipient mice. Four days post adoptive transfer donor T cells from perLN and mesLN were stained with eIF4E or isotype control antibodies as described above. T_Foxp3−_ (CD4^+^GFP^−^) and T_Foxp3+_ (CD4^+^GFP^+^) cells were analyzed for eIF4E expression and eFluor 670 levels by FACS.

### Western blot analysis

Cell lysates were prepared from activated T_Foxp3+_ and T_Foxp3−_ cells, and western blotting was carried out as previously described [28] using 25 µg of protein per sample. Antibodies against eIF4E (BD Biosciences, Mississauga, ON) and β-actin (AC-15, Sigma, St. Louis, MO) were used at a 1∶1000 and 1∶5000 dilutions, respectively. Antibodies against Anapc4 (Bethyl Laboratories, Montgomery, TX), cyclin-E1 (Abcam, Cambridge, MA), and cyclin-D3 (Cell Signaling Technology, Danvers, MA) were used at 1∶1000 dilution.

## Supporting Information

Figure S1 The translational signature contrasting activated CD4^+^ cell subsets is unique as compared to previous steady-state mRNA signatures. We compared the number of mRNAs that were significantly differentially translated (>3-fold translational regulation) and also showed >3-fold steady-state mRNA regulation. A low percentage overlap designates a translational signature that is previously uncharacterized while a high percentage overlap indicates that it is redundant with previous studies. Density scatter plots (a blue scale from light to dark represents increasing local density of data points; outliers are indicated as dots) comparing genome wide expression levels (log2 scale) between conditions studied in previous steady-state mRNA assessments of the T_Foxp3+_ phenotype. For each comparison the mRNAs that were identified as translationally more active in activated T_Foxp3+_ or T_Foxp3−_ cells (>3-fold difference) are indicated as red and yellow dots respectively. The dotted lines indicate a >3-fold difference in the density scatter plot. The % of the mRNAs that were identified as the activated T cell translational signature (>3-fold difference) that also showed a >3-fold difference in the comparison is shown for each direction of regulation. Act: activated cells; Act TGFbeta: activated in the presence of TGFβ; LN: lymph nodes; LP: lamina propria; hi: high; lo: low; IL-2: cells were isolated from mice treated with IL-2.
(EPS)

Figure S2 The translational signature in activated CD4^+^ cells does not overlap with previous steady-state mRNA signatures. We compared the number of mRNAs that were significantly differentially translated (>3-fold translational regulation) and also showed >3-fold steady-state mRNA regulation. A low percentage overlap designates a translational signature that is previously uncharacterized while a high percentage overlap indicates that it is redundant with previous studies. Shown are 7 density scatter plots (a blue scale from light to dark represents increasing local density of data points; outliers are indicated as dots) comparing conditions studied in previous steady-state mRNA assessments of the T_Foxp3+_ phenotype. For each comparison the mRNAs that were identified as translationally more active in activated T_Foxp3+_ or T_Foxp3−_ cells in the present study (>3-fold difference) are indicated as red and yellow points respectively. The dotted lines indicate a >3-fold difference in the density scatter plot. The % of the mRNAs that were identified as the activated T cell translational signature (>3-fold difference) that also showed a >3-fold difference in the comparison is shown for each direction of regulation. Thy: thymus, hi: high; lo: low; Homeo conv: homeostatically converted through injection of T_Foxp3−_ cells into lymphopenic hosts; DEC-pept conv: antigen-specific conversion through injection of DEC205 specific T_Foxp3−_ cells into immuno-competent hosts followed by injection of the DEC205 peptide.
(EPS)

Figure S3 The translational signature in *ex vivo* CD4^+^ T cells is too small for efficient comparisons to previous steady-state mRNA signatures. Shown are 21 density scatter plots (a blue scale from light to dark represents increasing local density of data points; outliers are indicated as dots) comparing conditions studied in previous steady-state RNA assessments of the T_Foxp3+_ phenotype. For each comparison the mRNAs from the T cell *ex vivo* translational signature (>2 fold difference) are indicated. The dotted lines indicate a >2 fold difference in the density scatter plot. The % of the mRNAs that were identified as the *ex vivo* T cell translational signature (>2 fold difference) that also showed a >2 fold difference in the comparison is shown for each direction of regulation. Act: activated; LN: lymph nodes; LP: lamina propria; hi: high; lo: low; IL-2: cells were isolated from mice treated with IL-2; ko: knock out; Thy: thymus; Homeo conv: homeostatically converted through injection of T_Foxp3−_ cells into lymphopenic hosts; DEC pept conv: antigen specific conversion through injection of DEC205 specific T_Foxp3−_ cells into immuno-competent hosts followed by injection of the DEC205 peptide.
(EPS)

Figure S4 Chemical structure of mRNA cap analogues. The selective inhibitors of mRNA cap structure-binding to eIF4E are shown: (Top) 4ei-1, a prodrug (pronucleotide phosphoramidate) of 7Bn-GMP (K_d_ of 0.80 µM); (Bottom) 4ei-4, a control prodrug of 7Me-GMP, which has a 10- fold lower affinity for eIF4E than 7-Bn-GMP (K_d_ = 7.5 µM).
(EPS)

Figure S5 Effect of 4ei-1 inhibitor on CD25 expression and viability following T_Foxp3−_ cell activation. (a) T_Foxp3−_ (left) and T_Foxp3+_ (right) cells were IL-2/TCR activated in the presence of increasing concentrations of 4ei-1 or 4ei-4. Cell viability was analyzed by flow cytometry using an eFluor780 Fixable Viability Dye exclusion assay after 72 h of culture. The percentage of viable cells is shown for each condition. (b) The effect of 4ei-1 and 4ei-4 on CD25 expression was analyzed by FACS on total CD4^+^ T cells activated as described above in the presence of increasing concentrations of 4ei-1 or 4ei-4. Shown is the mean fluorescence intensity (MFI) for CD25 in each condition.
(EPS)

Figure S6 Quantification of eIF4E protein level using flow cytometry. eFluor 670-labeled T_Foxp3−_ or T_Foxp3+_ cells were IL-2/TCR activated for the indicated time and analyzed for eIF4E expression using flow cytometry. Representative dot plots (n = 2) show T_Foxp3−_ and T_Foxp3+_ cell proliferation relative to eIF4E expression (left panel). Stainings with an isotype control are shown as contour plots. Quantification of eIF4E expression is shown as Δ (eIF4E *vs.* isotype control) mean fluorescent intensity (MFI) (right panel).
(EPS)

Figure S7 Foxo3 is translationally activated in activated T_Foxp3+_ cells. Anota analysis of translational activity. Shown is the cytosolic mRNA level (x-axis) vs. the polysome-associated mRNA level (y-axis) for each of the conditions analyzed; T_Foxp3+_ N (blue) and T_Foxp3−_ N (red) – *ex vivo* cells; T_Foxp3+_ 36 h (green) and T_Foxp3−_ 36 h (black) - activated cells. The lines indicate the regression lines used by anota to correct the polysome-associated mRNA level for the cytosolic mRNA level.
(EPS)

Table S1 Biological functions enriched among encoded proteins in the eIF4E-sensitive module.
(DOC)

## References

[1] Schwanhausser B, Busse D, Li N, Dittmar G, Schuchhardt J, et al (2011) Global quantification of mammalian gene expression control. Nature 473: 337–342. 21593866 10.1038/nature10098

[2] Vogel C, Abreu Rde S, Ko D, Le SY, Shapiro BA, et al (2010) Sequence signatures and mRNA concentration can explain two-thirds of protein abundance variation in a human cell line. Mol Syst Biol 6: 400. 20739923 10.1038/msb.2010.59 PMC2947365

[3] Gygi SP, Rochon Y, Franza BR, Aebersold R (1999) Correlation between protein and mRNA abundance in yeast. Mol Cell Biol 19: 1720–1730. 10022859 10.1128/mcb.19.3.1720 PMC83965

[4] Lu R, Markowetz F, Unwin RD, Leek JT, Airoldi EM, et al (2009) Systems-level dynamic analyses of fate change in murine embryonic stem cells. Nature 462: 358–362. 19924215 10.1038/nature08575 PMC3199216

[5] Persson O, Brynnel U, Levander F, Widegren B, Salford LG, et al (2009) Proteomic expression analysis and comparison of protein and mRNA expression profiles in human malignant gliomas. Proteomics Clin Appl 3: 83–94. 21136938 10.1002/prca.200800086

[6] Washburn MP, Koller A, Oshiro G, Ulaszek RR, Plouffe D, et al (2003) Protein pathway and complex clustering of correlated mRNA and protein expression analyses in Saccharomyces cerevisiae. Proc Natl Acad Sci U S A 100: 3107–3112. 12626741 10.1073/pnas.0634629100 PMC152254

[7] Wong WF, Kohu K, Chiba T, Sato T, Satake M (2011) Interplay of transcription factors in T cell differentiation and function: the role of Runx. Immunology 132: 157–164. 21091910 10.1111/j.1365-2567.2010.03381.x PMC3050439

[8] Sakaguchi S, Ono M, Setoguchi R, Yagi H, Hori S, et al (2006) Foxp3+ CD25+ CD4+ natural regulatory T cells in dominant self-tolerance and autoimmune disease. Immunol Rev 212: 8–27. 16903903 10.1111/j.0105-2896.2006.00427.x

[9] Sakaguchi S, Yamaguchi T, Nomura T, Ono M (2008) Regulatory T cells and immune tolerance. Cell 133: 775–787. 18510923 10.1016/j.cell.2008.05.009

[10] Zhu J, Paul WE (2008) CD4 T cells: fates, functions, and faults. Blood 112: 1557–1569. 18725574 10.1182/blood-2008-05-078154 PMC2518872

[11] Feuerer M, Hill JA, Kretschmer K, von Boehmer H, Mathis D, et al (2010) Genomic definition of multiple ex vivo regulatory T cell subphenotypes. Proc Natl Acad Sci U S A 107: 5919–5924. 20231436 10.1073/pnas.1002006107 PMC2851866

[12] Fontenot JD, Rasmussen JP, Gavin MA, Rudensky AY (2005) A function for interleukin 2 in Foxp3-expressing regulatory T cells. Nat Immunol 6: 1142–1151. 16227984 10.1038/ni1263

[13] Fontenot JD, Rasmussen JP, Williams LM, Dooley JL, Farr AG, et al (2005) Regulatory T cell lineage specification by the forkhead transcription factor foxp3. Immunity 22: 329–341. 15780990 10.1016/j.immuni.2005.01.016

[14] Gavin MA, Rasmussen JP, Fontenot JD, Vasta V, Manganiello VC, et al (2007) Foxp3-dependent programme of regulatory T cell differentiation. Nature 445: 771–775. 17220874 10.1038/nature05543

[15] Hill JA, Feuerer M, Tash K, Haxhinasto S, Perez J, et al (2007) Foxp3 transcription-factor-dependent and -independent regulation of the regulatory T cell transcriptional signature. Immunity 27: 786–800. 18024188 10.1016/j.immuni.2007.09.010

[16] Grolleau A, Bowman J, Pradet-Balade B, Puravs E, Hanash S, et al (2002) Global and specific translational control by rapamycin in T cells uncovered by microarrays and proteomics. J Biol Chem 277: 22175–22184. 11943782 10.1074/jbc.M202014200

[17] Garcia-Sanz JA, Mikulits W, Livingstone A, Lefkovits I, Mullner EW (1998) Translational control: a general mechanism for gene regulation during T cell activation. FASEB J 12: 299–306. 9506473 10.1096/fasebj.12.3.299

[18] Mikulits W, Pradet-Balade B, Habermann B, Beug H, Garcia-Sanz JA, et al (2000) Isolation of translationally controlled mRNAs by differential screening. FASEB J 14: 1641–1652. 10928999 10.1096/fj.14.11.1641

[19] Sonenberg N, Hinnebusch AG (2009) Regulation of translation initiation in eukaryotes: mechanisms and biological targets. Cell 136: 731–745. 19239892 10.1016/j.cell.2009.01.042 PMC3610329

[20] Larsson O, Sonenberg N, Nadon R (2010) Identification of differential translation in genome wide studies. Proc Natl Acad Sci U S A 107 50: 21487–21492. 21115840 10.1073/pnas.1006821107 PMC3003104

[21] Larsson O, Sonenberg N, Nadon R (2011) anota: analysis of differential translation in genome wide studies. Bioinformatics 27 10: 1440–1. 21422072 10.1093/bioinformatics/btr146

[22] Feuerer M, Hill JA, Mathis D, Benoist C (2009) Foxp3+ regulatory T cells: differentiation, specification, subphenotypes. Nat Immunol 10: 689–695. 19536194 10.1038/ni.1760

[23] Keene JD (2007) RNA regulons: coordination of post-transcriptional events. Nat Rev Genet 8: 533–543. 17572691 10.1038/nrg2111

[24] Mukherjee N, Lager PJ, Friedersdorf MB, Thompson MA, Keene JD (2009) Coordinated posttranscriptional mRNA population dynamics during T cell activation. Mol Syst Biol 5: 288. 19638969 10.1038/msb.2009.44 PMC2724974

[25] Hogan DJ, Riordan DP, Gerber AP, Herschlag D, Brown PO (2008) Diverse RNA-binding proteins interact with functionally related sets of RNAs, suggesting an extensive regulatory system. PLoS Biol 6: e255 doi:10.1371/journal.pbio.0060255. 18959479 10.1371/journal.pbio.0060255 PMC2573929

[26] Luo W, Friedman MS, Shedden K, Hankenson KD, Woolf PJ (2009) GAGE: generally applicable gene set enrichment for pathway analysis. BMC Bioinformatics 10: 161. 19473525 10.1186/1471-2105-10-161 PMC2696452

[27] Larsson O, Li S, Issaenko OA, Avdulov S, Peterson M, et al (2007) Eukaryotic translation initiation factor 4E induced progression of primary human mammary epithelial cells along the cancer pathway is associated with targeted translational deregulation of oncogenic drivers and inhibitors. Cancer Res 67: 6814–6824. 17638893 10.1158/0008-5472.CAN-07-0752

[28] Mamane Y, Petroulakis E, Martineau Y, Sato TA, Larsson O, et al (2007) Epigenetic activation of a subset of mRNAs by eIF4E explains its effects on cell proliferation. PLoS ONE 2: e242 doi:10.1371/journal.pone.0000242. 17311107 10.1371/journal.pone.0000242 PMC1797416

[29] Dowling RJ, Topisirovic I, Alain T, Bidinosti M, Fonseca BD, et al (2010) mTORC1-mediated cell proliferation, but not cell growth, controlled by the 4E-BPs. Science 328: 1172–1176. 20508131 10.1126/science.1187532 PMC2893390

[30] Larsson O, Perlman DM, Fan D, Reilly CS, Peterson M, et al (2006) Apoptosis resistance downstream of eIF4E: posttranscriptional activation of an anti-apoptotic transcript carrying a consensus hairpin structure. Nucleic Acids Res 34: 4375–4386. 16936314 10.1093/nar/gkl558 PMC1636353

[31] Colina R, Costa-Mattioli M, Dowling RJ, Jaramillo M, Tai LH, et al (2008) Translational control of the innate immune response through IRF-7. Nature 452: 323–328. 18272964 10.1038/nature06730

[32] Kim YY, Von Weymarn L, Larsson O, Fan D, Underwood JM, et al (2009) Eukaryotic initiation factor 4E binding protein family of proteins: sentinels at a translational control checkpoint in lung tumor defense. Cancer Res 69: 8455–8462. 19843855 10.1158/0008-5472.CAN-09-1923 PMC2805259

[33] Bour-Jordan H, Bluestone JA (2009) Regulating the regulators: costimulatory signals control the homeostasis and function of regulatory T cells. Immunol Rev 229: 41–66. 19426214 10.1111/j.1600-065X.2009.00775.x PMC2714548

[34] Thornton AM, Shevach EM (1998) CD4+CD25+ immunoregulatory T cells suppress polyclonal T cell activation in vitro by inhibiting interleukin 2 production. J Exp Med 188: 287–296. 9670041 10.1084/jem.188.2.287 PMC2212461

[35] Ghosh B, Benyumov AO, Ghosh P, Jia Y, Avdulov S, et al (2009) Nontoxic chemical interdiction of the epithelial-to-mesenchymal transition by targeting cap-dependent translation. ACS Chem Biol 4: 367–377. 19351181 10.1021/cb9000475 PMC2796976

[36] Delgoffe GM, Kole TP, Zheng Y, Zarek PE, Matthews KL, et al (2009) The mTOR kinase differentially regulates effector and regulatory T cell lineage commitment. Immunity 30: 832–844. 19538929 10.1016/j.immuni.2009.04.014 PMC2768135

[37] Sauer S, Bruno L, Hertweck A, Finlay D, Leleu M, et al (2008) T cell receptor signaling controls Foxp3 expression via PI3K, Akt, and mTOR. Proc Natl Acad Sci U S A 105: 7797–7802. 18509048 10.1073/pnas.0800928105 PMC2409380

[38] Nikolcheva T, Pyronnet S, Chou SY, Sonenberg N, Song A, et al (2002) A translational rheostat for RFLAT-1 regulates RANTES expression in T lymphocytes. J Clin Invest 110: 119–126. 12093895 10.1172/JCI15336 PMC151028

[39] Villarino AV, Katzman SD, Gallo E, Miller O, Jiang S, et al (2011) Posttranscriptional silencing of effector cytokine mRNA underlies the anergic phenotype of self-reactive T cells. Immunity 34: 50–60. 21236706 10.1016/j.immuni.2010.12.014 PMC3955755

[40] Mao X, Green JM, Safer B, Lindsten T, Frederickson RM, et al (1992) Regulation of translation initiation factor gene expression during human T cell activation. J Biol Chem 267: 20444–20450. 1400363

[41] Fang L, Wang H, Zhou L, Yu D (2011) FOXO3a reactivation mediates the synergistic cytotoxic effects of rapamycin and cisplatin in oral squamous cell carcinoma cells. Toxicol Appl Pharmacol 251: 8–15. 21092744 10.1016/j.taap.2010.11.007

[42] Huang H, Regan KM, Wang F, Wang D, Smith DI, et al (2005) Skp2 inhibits FOXO1 in tumor suppression through ubiquitin-mediated degradation. Proc Natl Acad Sci U S A 102: 1649–1654. 15668399 10.1073/pnas.0406789102 PMC545492

[43] Ouyang W, Beckett O, Ma Q, Paik JH, DePinho RA, et al (2010) Foxo proteins cooperatively control the differentiation of Foxp3+ regulatory T cells. Nat Immunol 11: 618–627. 20467422 10.1038/ni.1884

[44] Zhou L, Chong MM, Littman DR (2009) Plasticity of CD4+ T cell lineage differentiation. Immunity 30: 646–655. 19464987 10.1016/j.immuni.2009.05.001

[45] Dai M, Wang P, Boyd AD, Kostov G, Athey B, et al (2005) Evolving gene/transcript definitions significantly alter the interpretation of GeneChip data. Nucleic Acids Res 33: e175. 16284200 10.1093/nar/gni179 PMC1283542

[46] Boyle EI, Weng S, Gollub J, Jin H, Botstein D, et al (2004) TermFinder–open source software for accessing Gene Ontology information and finding significantly enriched Gene Ontology terms associated with a list of genes. Bioinformatics 20: 3710–3715. 15297299 10.1093/bioinformatics/bth456 PMC3037731

