# Supplementary material for: Distinct Translational Control in CD4+ T Cell Subsets
Source: PLoS Genet. 2013 May 2;9(5):e1003494. doi: 10.1371/journal.pgen.1003494 (PMC3642068; doi:10.1371/journal.pgen.1003494)
Supplement: Table S1 — Biological functions enriched among encoded proteins in the eIF4E-sensitive module. (DOC) [file pgen.1003494.s008.doc]

| **Cellular functions** | **Adjusted p-values** |
| --- | --- |
| cell cycle | 2.44E-07 |
| cell cycle phase | 2.20E-05 |
| mitotic cell cycle | 2.47E-05 |
| cell cycle process | 4.78E-05 |
| M phase | 5.46E-05 |
| mitosis | 7.32E-05 |
| M phase of mitotic cell cycle | 7.32E-05 |
| nuclear division | 7.32E-05 |
| organelle fission | 8.60E-05 |
| cell division | 1.23E-03 |
| ubiquitin dependent protein catabolic process | 5.74E-03 |
| modification dependent macromolecule catabolic process | 5.79E-03 |
| modification dependent protein catabolic process | 5.79E-03 |
| proteolysis involved in cellular protein catabolic process | 7.44E-03 |
| cellular protein catabolic process | 7.59E-03 |
| chromosome organization | 9.06E-03 |
| protein catabolic process | 9.06E-03 |

Table S1. Analysis of biological processes (from the Gene Ontology Consortium) enriched in the eIF4E profile (FDR<1%). The p-values for enrichment were adjusted for multiple testing.
